# Minimization of thermal deformation in crystal optics for high-repetition-rate FEL

**DOI:** 10.1107/S1600577525011658

**Published:** 2026-02-09

**Authors:** Lin Zhang, Jerome Hastings, Zhirong Huang, Jean-Pierre Torras, Diling Zhu

**Affiliations:** a LCLS SLAC National Accelerator Laboratory 2575 Sand Hill Road Menlo Park CA94025 USA; Utrecht University, The Netherlands

**Keywords:** free-electron laser, X-ray crystal optics, pulse-by-pulse transient thermal deformation, liquid nitro­gen cooling, second-order correction

## Abstract

Minimizing thermal deformation in X-ray crystal optics is crucial for preserving coherence and wavefront in high-repetition-rate free-electron lasers (FELs). This study presents two approaches to reduce pulse-by-pulse transient thermal deformation in diamond crystals used in cavity-based X-ray FELs (CBXFELs): (i) cryogenic cooling with liquid nitro­gen (LN_2_), and (ii) second-order correction via focusing optics. We revisit the temperature-dependent thermal-mechanical properties of diamond and silicon, and implement a finite-element analysis method to accelerate convergence to a quasi-steady-state regime. Results show that LN_2_-cooled diamond crystals meet the stringent deformation requirement of less than 15 pm RMS for the pulse at the mJ scale at 1 MHz repetition frequency, and up to 1.5 mJ for 100 kHz. Second-order correction by using focusing elements within the cavity can reduce the impact of thermal deformation for both LN_2_ and water cooling.

## Abstract 2

Thermal deformation in X-ray crystal optics in high-repetition-rate X-ray free-electron lasers can be effectively reduced by cryogenic cooling with liquid nitro­gen, and possibly by second-order correction via focusing optics.

## 1. Introduction

Hard X-ray free-electron lasers (XFELs) operating under the self-amplified spontaneous emission (SASE) mode are quite monochromatic; the energy bandwidth Δ*e*_ph_/*e*_ph_ is of the order of ∼0.001 (Huang & Ruth, 2006 ▸). This level of monochromaticity of the SASE XFEL was sufficient for many early science applications but requires further monochromatization by crystal optics for spectroscopy and diffraction applications (Zhu *et al.*, 2014 ▸). This leads to significant reduction in usable X-ray pulse energy and flux. Demands for higher spectral brightness from experiments calls for new XFEL concepts such as CBXFELs (cavity-based X-ray free-electron lasers) which also require the use of high-quality crystal-based optics (Rysov *et al.*, 2019 ▸; Huang & Ruth, 2006 ▸; Kim *et al.*, 2008 ▸; Marcus *et al.*, 2020 ▸; Rauer *et al.*, 2023 ▸; Margraf *et al.*, 2023 ▸; Zhang *et al.*, 2023 ▸). Single crystal silicon has been widely used in optics for storage-ring based synchrotron radiation facilities. To cope with the extreme high peak power of the XFEL, diamond crystal optics have become increasingly popular (Zhu *et al.*, 2014 ▸). High-repetition-rate XFELs promise to deliver orders of magnitude higher average power (up to several hundreds of Watts) than the currently operating low-repetition-rate XFELs. To preserve the excellent beam properties such as brightness, coherence, and the extremely short pulse duration, thermal deformation of the optics needs to be minimized to an unprecedented level of a few tens of picometres in RMS figure error (Zhang *et al.*, 2023 ▸). Therefore, active cooling of the crystal optics becomes mandatory. Compared with storage-ring based synchrotron radiation light sources, XFEL light sources deliver ultra-short pulses with variable repetition rates. The time structure of the XFEL and transient response of the crystal optics raise new challenges and questions in the application of crystal optics.

The relation between thermal deformation of X-ray optics and constant isotropic material properties can be expressed as (Zhang *et al.*, 2014 ▸; Zhang, 2018 ▸)
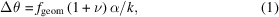
where Δθ is the thermal slope error; ν, α, *k* are, respectively, Poisson’s ratio, the thermal expansion coefficient, and thermal conductivity; *f*_geom_ is a dimensionless geometrical function depending on the optics and cooling geometry, and power distribution. This equation indicates that thermal deformation can be minimized in two ways: (i) optimizing the cooling design and geometrical shape of the optics (Zhang *et al.*, 2013*a* ▸; Zhang *et al.*, 2015 ▸), and (ii) material selection (or operating temperature) to achieve good thermal mechanical properties: high thermal conductivity and low thermal expansion coefficient. For a crystal monochromator, liquid-nitro­gen (LN_2_) cooling is the most effective way to limit thermal deformations. LN_2_-cooled silicon crystals have been widely used with great success at many synchrotron light sources (Marot *et al.*, 1992 ▸; Rogers *et al.*, 1995 ▸; Lee *et al.*, 1995 ▸; Marot, 1995 ▸; also see the reviews by Bilderback *et al.*, 2000 ▸; Mochizuki *et al.*, 2001 ▸; Zhang *et al.*, 2003 ▸; Chumakov *et al.*, 2004 ▸). Numerous studies have been carried out to assess the performance limits of cryogenically cooled silicon monochromators both by ﬁnite-element analysis (FEA) modelling and experimental testing (Zhang, 1993 ▸; Lee *et al.*, 2000 ▸, 2001 ▸; Tajiri *et al.*, 2001 ▸; Zhang *et al.*, 2003 ▸, 2013*b* ▸; Chumakov *et al.*, 2014 ▸; Huang *et al.*, 2014 ▸).

Pulse-by-pulse transient thermal deformation in water-cooled diamond crystal optics under high-repetition-rate FEL has been studied (Bahns *et al.*, 2023 ▸; Zhang *et al.*, 2025 ▸). The CBXFEL system that serves as the background of this study is composed of four diamond crystals forming a 300 m-long round-trip cavity, with a round-trip time of 1 µs. The narrow bandwidth XFEL pulse recirculates as a seed in the cavity. The number of recirculation cycles of this seed is 2, 10, and 20 for a FEL repetition rate of 500, 100, and 50 kHz, determined by electron bunch repetition rate. The outcoupling crystal absorbs a fraction of output FEL power and thus is the most critical crystal in the cavity in terms of thermal deformation. To limit this thermal deformation to less than 20 pm for the wavefront preservation requirement, the FEL pulse energy should be limited to less than 100 µJ at 100 kHz repetition rate and 50 µJ at 500 kHz repetition rate. However, the LCLS, LCLS-II, and LCLS-II-HE FEL can deliver up to several mJ scale pulse energies. To transport and manipulate such high pulse energy FEL beams, the thermal deformation of the crystal should be significantly reduced.

This paper studies two methods to minimize the thermal deformation of the crystal for high-repetition-rate FEL: (i) LN_2_ cooling, (ii) second-order compensation by using focusing elements. First, we revisit temperature dependent material properties such as thermal conductivity, thermal expansion coefficient, and heat capacity of diamond and silicon crystals. We propose a simulation technique to accelerate the convergence of pulse-by-pulse transient analysis. Finally, we focus on the performance improvement of LN_2_ cooling, and second-order compensation by using focusing elements.

## 2. Temperature dependent thermal mechanical properties of silicon and diamond

Historically, silicon has been used in X-ray optics for both crystal monochromators and mirror substrates. However, synthetic HTHP single crystal diamond is increasingly being considered for crystal monochomators, especially for XFELs. The thermal conductivity *k* and thermal expansion coefficient α are two key material properties that affect the thermal deformation of X-ray optics. The thermal deformation increases with (1 + ν)*a*/*k* [equation (1)]. The thermal transient response of the X-ray optics, under pulse-by-pulse XFEL power load, also depends on heat capacity *c*_p_ and density ρ. In addition, Young’s modulus *E* also affects transient deformation and acoustic waves (Zhang *et al.*, 2025 ▸). The thermal conductivity *k*, thermal expansion coefficient α, and heat capacity *c*_p_ of the silicon and diamond are strongly temperature dependent. On the other hand, Young’s modulus *E*, density ρ, and Poisson’s ratio ν are quite constant in the temperature range 80 to 500 K.

### 2.1. Thermal conductivity

**Figure 1:**
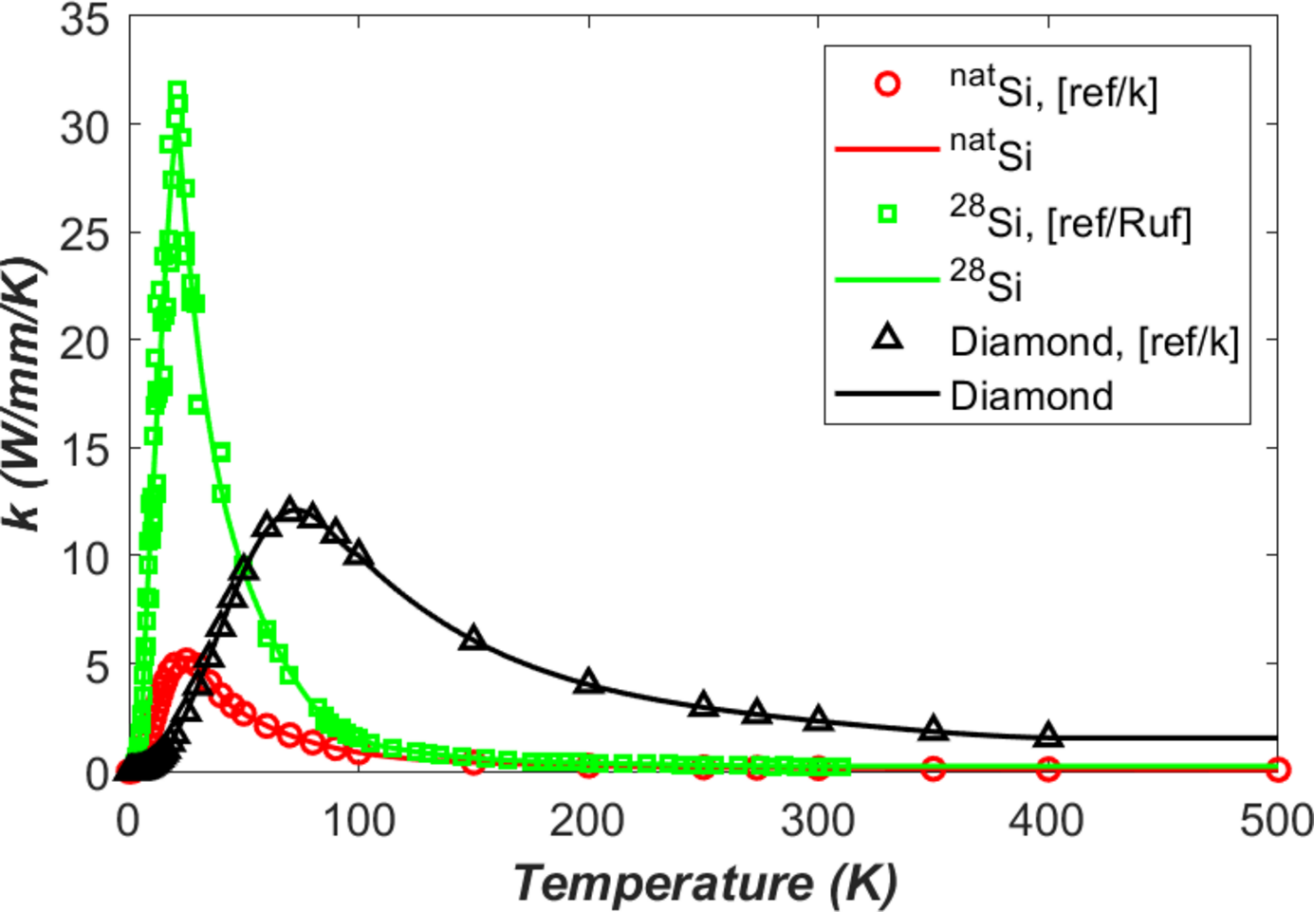
Thermal conductivity of natural silicon, single-isotope silicon-28 (99.9%), and diamond IIa. The continuous lines are curve fit results of the data represented by small squares, circles, and triangles.

The thermal conductivity *k* versus temperature is depicted in Fig. 1 ▸ for type IIa single crystal diamond, natural silicon, and single-isotope silicon-28 (99.9%). Type IIa single crystal diamonds are the most chemically pure diamonds and have the highest thermal conductivity beside isotropically pure varieties. In the plots, the data represented by small circles for natural silicon and by small triangles for diamond IIa are the recommended values (Touloukian, Powell *et al.*, 1970 ▸), and the data represented by small squares for single-isotope silicon-28 crystal are measured values (Ruf *et al.*, 2000 ▸). The continuous lines are curve fit results of the data. The polynomials used for curve fitting are given in Appendix *A*1[App appa].

The maximum of thermal conductivity of single-isotope silicon-28 is significantly higher than that of natural silicon. At very low temperatures, heat transfer is mainly due to phonon boundary scattering, and the thermal conductivity *k* of the crystals is proportional to the temperature *T*^3^ according to the Debye theory (see, for example, Van Sciver, 1986 ▸; Ruf *et al.*, 2000 ▸).

The thermal conductivity of the silicon and diamond crystal is strongly temperature dependent. The shape of the thermal conductivity versus temperature curve of the three crystals is similar: the thermal conductivity increases from zero with *T*^3^ at very low temperature, then continuously increases with temperature, reaching a peak of *k* = 5.2 W mm^−1^ K^−1^ at 25 K for natural silicon, *k* = 30 W mm^−1^ K^−1^ at 21 K for single-isotope silicon-28, and *k* = 12 W mm^−1^ K^−1^ at about 70 K for diamond IIa. The peak thermal conductivity values of the crystals are much higher than at room temperature, 124 times for single-isotope silicon-28, 36 times for natural silicon, and 5.2 times for diamond IIa. Above 50 K, the diamond IIa crystal offers the highest thermal conductivity. The superiority of diamond crystal at room temperature is undeniable. Below 50 K, the single-isotope silicon-28 gives the highest thermal conductivity; the peak value (*k* = 30 W mm^−1^ K^−1^) is 2.5 times that of the diamond IIa crystal, and 5.8 times that of the natural silicon crystal. As a perfect single crystal, the single-isotope silicon-28 could be an excellent candidate for monochromators cooled to low temperatures down to 20 K.

### 2.2. Thermal expansion coefficient

**Figure 2:**
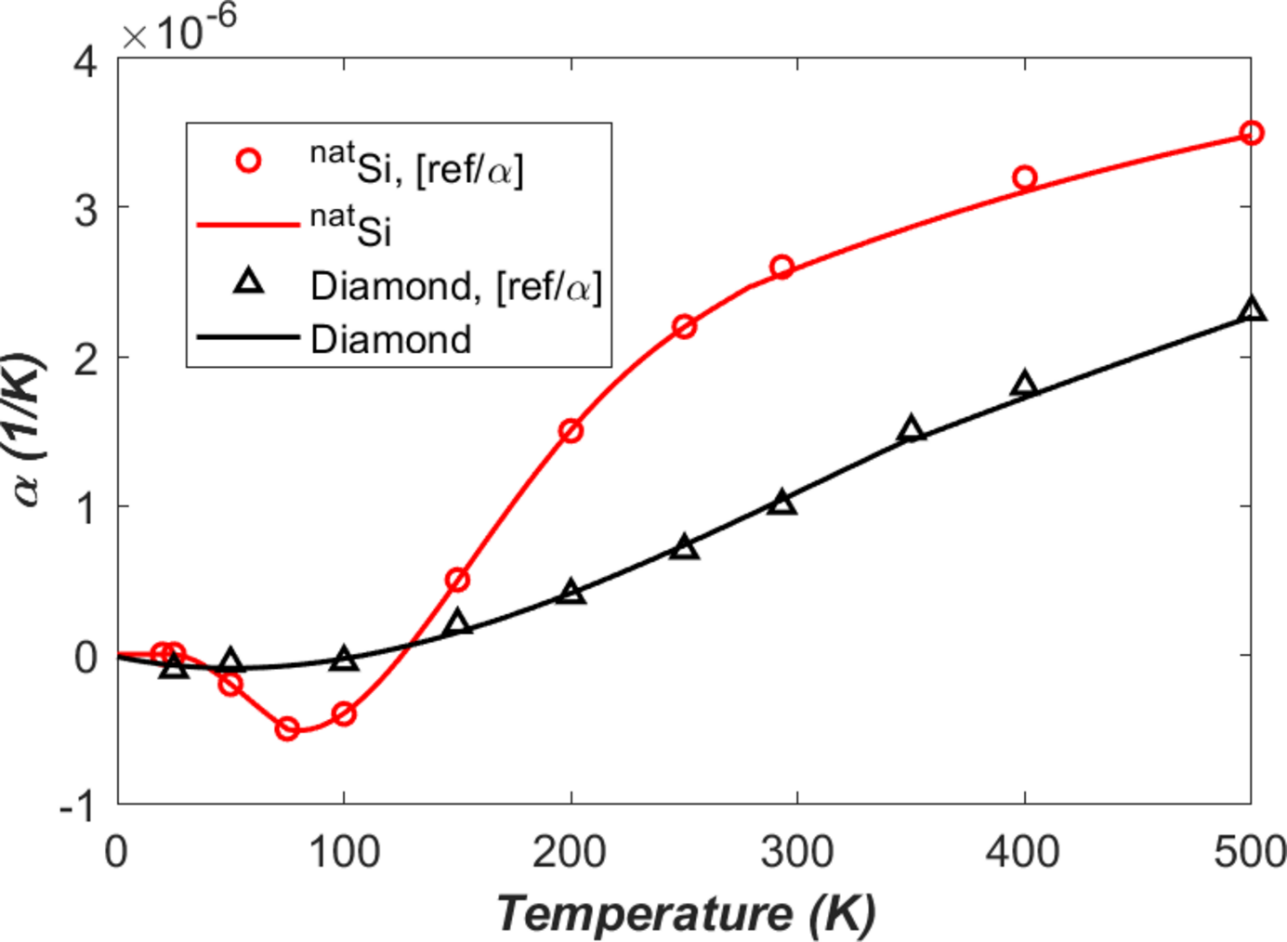
Thermal expansion coefficient α versus temperature of silicon and diamond. The continuous lines are curve fit results of the data represented by small circles and triangles.

The thermal expansion coefficient α versus temperature is shown in Fig. 2 ▸ for (natural) silicon and diamond. In the graph, the data represented by small circles for silicon and by small triangles for diamond are the values recommended by Touloukian, Kirby *et al.* (1970 ▸). Natural silicon is composed of 92% silicon-28; the thermal expansion coefficient of the single-isotope silicon-28 and the natural silicon are not significantly different from each other. The continuous lines are curve fit results of the data. Curve-fitted polynomials are given in Appendix *A*2[App appa].

The thermal expansion coefficient α data (Touloukian, Kirby *et al.*, 1970 ▸) are in the temperature ranges 20–1600 K for silicon and 25–1600 K for diamond. According to physical analysis (Van Sciver, 1986 ▸), the thermal expansion coefficient at low temperatures near zero K is far from linear and approaches absolute zero with zero slope. The silicon data (Touloukian, Kirby *et al.*, 1970 ▸) show α = 0 at the two lowest temperatures *T* = 20 and 25 K. Below 20 K, the thermal expansion coefficient α of the silicon is naturally set to be zero. For diamond, the data from Touloukian, Kirby *et al.* (1970 ▸) at the lowest temperature 25 K are not zero. As the thermal expansion coefficient at low temperature approaches absolute zero with zero slope, the thermal expansion coefficient α of the diamond is therefore set to be zero below 5 K.

Like the thermal conductivity, the thermal expansion coefficient α is also strongly temperature dependent. The value of α is negative when temperature *T* is lower than 125 K for silicon, and lower than 111 K for diamond (can be checked with fitted formula in Appendix *A*[App appa]). The thermal expansion coefficient of the diamond is very close to zero below 111 K. Above these temperatures the thermal expansion coefficient increases with temperature, especially for silicon.

**Figure 3:**
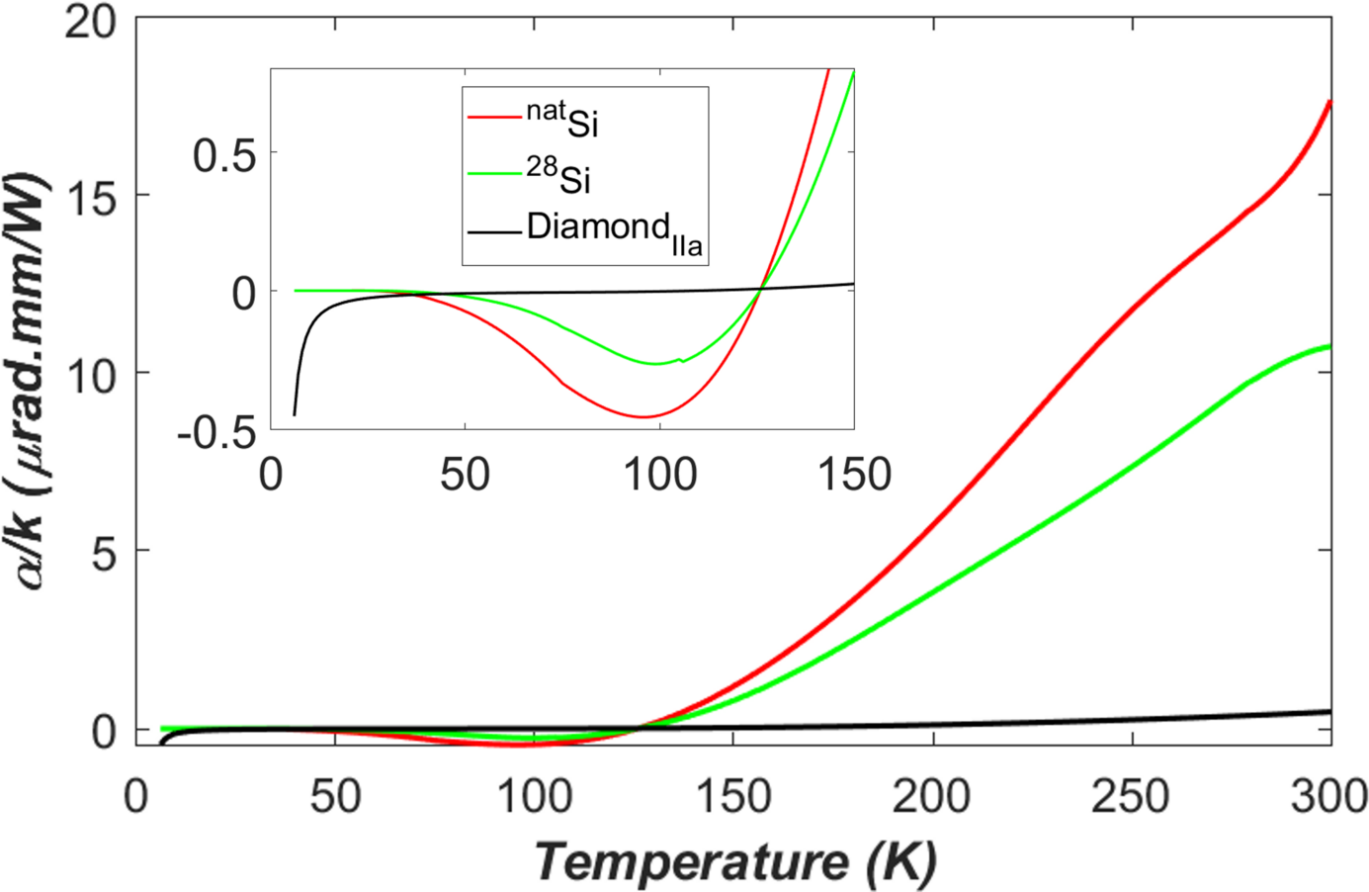
Ratio of thermal expansion coefficient and thermal conductivity α/κ versus temperature for the silicon and the diamond.

The ratio of thermal expansion coefficient and thermal conductivity α/κ versus temperature for the silicon and the diamond is plotted in Fig. 3 ▸. In general, this figure of merit α/κ for thermal deformation [see equation (1), and Freund *et al.* (1990 ▸)] of the diamond is flat and approaches zero. Therefore, diamond crystal is the best material for crystal monochromators in terms of thermal deformation. However, at very low temperatures, the natural silicon (*T* < 36 K) and single-isotope silicon-28 (*T* < 45 K) are better than diamond. The ideal working temperature range of the silicon crystal is between 20 and 25 K. To cool down to this temperature range, appropriate coolant, for example liquid helium, must be used. From the cooling power limitation, and cost effectiveness point of view, LN_2_ cooling is much more accessible than liquid helium cooling. Therefore, LN_2_ cooled diamond crystal can be an excellent option for high heat load XFEL monochromators.

### 2.3. Specific heat

**Figure 4:**
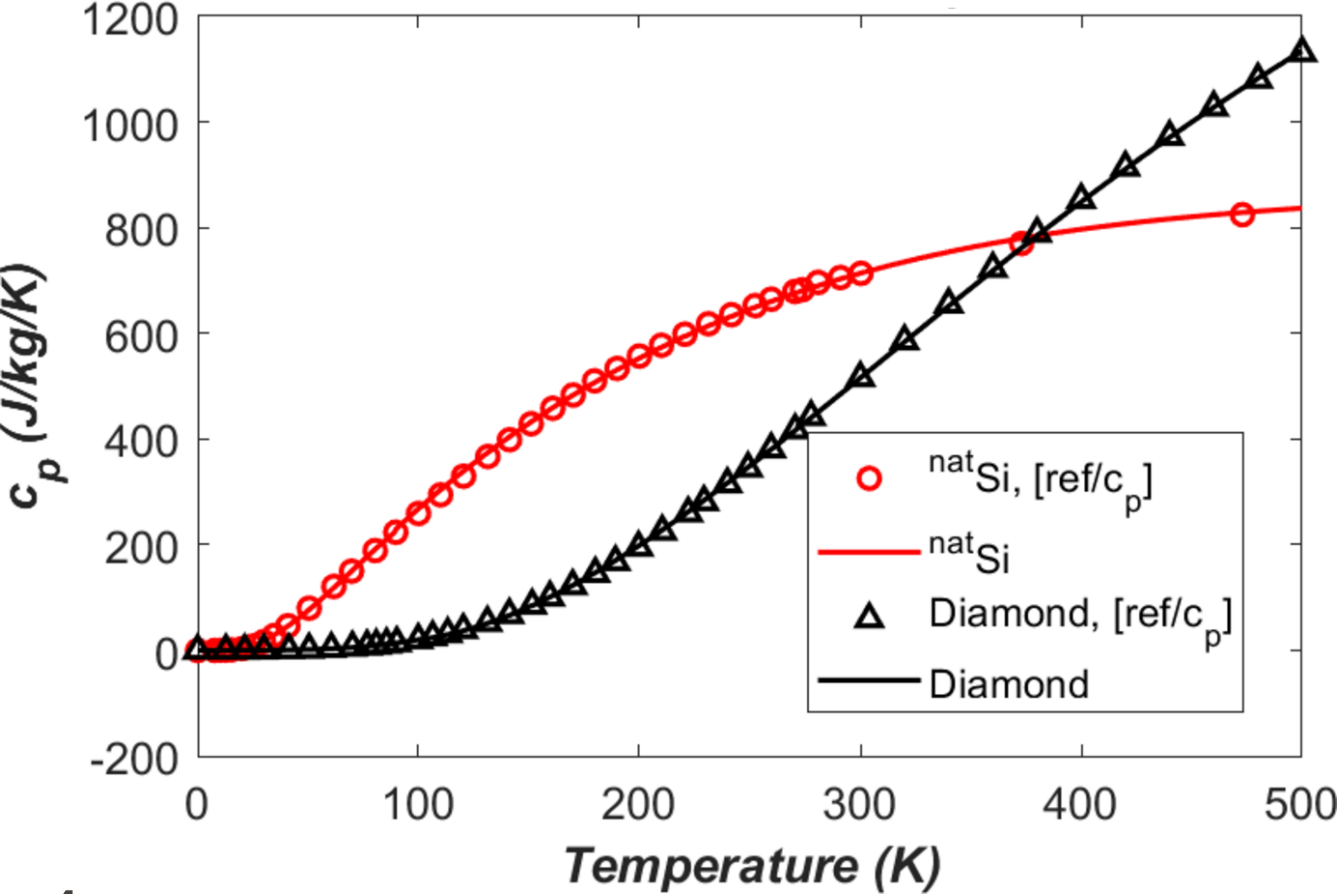
Specific heat *c*_p_ versus temperature of the silicon and the diamond. The continuous lines are curve fit results of the data represented by small circles and triangles.

To study the transient response of the monochromator crystal under pulse-by-pulse XFEL, the temperature dependent specific heat of the crystal is necessary in the transient analysis. Fig. 4 ▸ shows the specific heat *c*_p_ versus temperature for silicon and diamond. In the plots, the data represented by small circles for silicon and by small triangles for diamond are the recommended values by Touloukian, Buyco & Matter (1970 ▸). Hu *et al.* (2002 ▸) compared the low-temperature specific heat of single-isotope silicon-28 crystal with that of natural silicon through experimentation. Results in the temperature range 1–140 K show that the enhancement (about 10%) of specific heat by isotope purification is much less than that of the thermal conductivity. No data on the specific heat for single-isotope silicon-28 at temperatures higher than 140 K was found in the literature. Therefore, the data presented here for natural silicon will also be used for single-isotope silicon-28 in the finite element modelling. The continuous lines are curve fit results of the data represented by the small circles and triangles. The curve-fitted polynomials are given in Appendix *A*3[App appa].

The thermal diffusivity *D* can be calculated from the heat capacity, thermal conductivity, and density of the crystals as
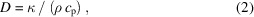
where ρ is the density of the material (ρ = 2.3 g cm^−3^ for silicon, ρ = 3.52 g cm^−3^ for diamond). The thermal diffusivity at lower temperature is much larger than at room temperature with the combined effects of temperature dependence of the thermal conductivity and specific heat.

The thermal diffusivity can be used to estimate the thermal diffusion time *τ*_th_. For example, in a length *L*, the thermal diffusion time is about
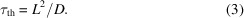
Table 1 ▸ gives this typical diffusion time for a 1 mm length of the three types of crystals at different temperatures. The thermal diffusivity of the three types of crystals at low temperature is much higher than at room temperature. Consequently, the thermal diffusion time in a length of 1 mm decreases from 11 ms at room temperature down to 0.29 ms at 77 K for natural silicon, from 6.8 ms to 0.12 ms for single-isotope silicon-28, and from 0.51 ms down to 0.0017 ms for diamond. The thermalization in the LN_2_ cooled crystals is much quicker than in the water-cooled crystals.

## 3. Transient thermal deformation modelling by FEA

**Figure 5:**
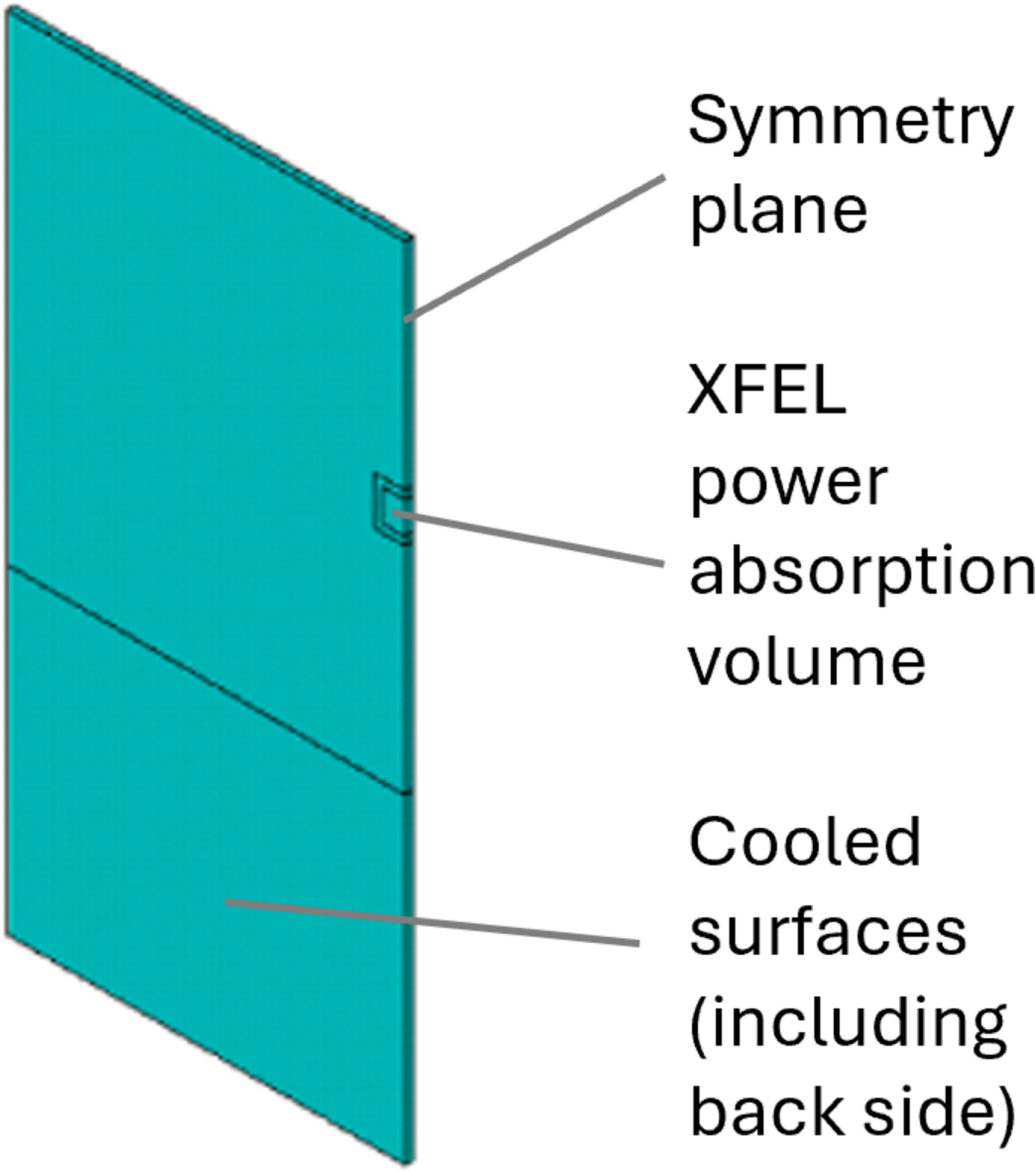
Finite element geometrical model of a half diamond crystal, symmetry condition on the right edge plane.

Pulse-by-pulse transient simulation of the crystal under XFEL is compute-intensive since the mesh size should be small enough, around the micrometre scale, to be consistent with ns scale time increments. A small, thin crystal can lead to a reasonable number of elements such that the simulation of a transient response to a quasi-steady-state can be performed in about one day^1^. In this study, we continue to consider the same thin diamond crystal C_1_ for CBXFEL as studied by Zhang *et al.* (2025 ▸). The diamond crystal is modelled as a thin rectangular prism (5 mm × 5 mm × 0.05 mm), with the lower 2 mm held on both sides by copper cooling blocks with indium foil interfaces. A geometrical model of the half crystal is shown in Fig. 5 ▸. The same thermal and mechanical boundary conditions are used in this study as Zhang *et al.* (2025 ▸), unless otherwise indicated for pulse energy and repetition frequency.

Other FEA parameters that are reused from Zhang *et al.* (2025 ▸) include: mesh size (down to 6 µm), time increments of mostly 1 ns, power loading pulse length of 1 ns, damping ratio of ζ = 0.5 (as we focus on temperature gradient related thermal deformation), and some constant material properties: density ρ = 2530 kg m^−3^, Young’s modulus *E* = 1000 GPa, and Poisson’s ratio ν = 0.1. However, temperature dependent thermal conductivity, thermal expansion coefficient, and specific heat capacity shown in Figs. 1 ▸, 2 ▸, and 4 ▸ will be used in this study. To quantify the thermal deformation, we will use the standard deviation (std) of the height error (vertical displacement *U*_*y*_) along the beam footprint axis on the crystal surface.

### 3.1. Accelerate the convergence to the quasi steady-state

The shape of the transient response curve of the X-ray optics under pulse-by-pulse XFEL is typically marked by a sharp increase in temperature or thermal deformation with arrival of the pulse, then followed by an exponential decay shortly after the pulse. With an accumulation of pulses, the average temperature level and thermal deformation increase with time until a certain time or number of pulses. The response curve pattern then becomes repetitive and is considered ‘quasi steady-state’. From the previous study (Zhang *et al.*, 2025 ▸) on the diamond crystal mentioned above, the time to reach this state is about 50 ms for temperature and 50 µs for thermal deformation. If the repetition rate is 1 MHz, 50 ms corresponds to 50000 pulses. The computing time for 169 XFEL pulses was 11 days. To reach quasi steady-state the computing time would be about ten years for temperature.

The previous study (Zhang *et al.*, 2025 ▸) showed that both temperature and thermal deformation at any repetition rate varies with time and oscillates around the results for the transient response with average XFEL power (called the CW case). This is true starting from the first XFEL pulse to quasi steady-state. The optics performance at this latter state is essential for beamline operation. To accelerate the transient response simulation to reach this quasi steady-state, we propose starting the transient FEA simulation using the steady-state simulation results with average XFEL power (CW steady-state) as the initial conditions both for temperature and displacement. To directly compare with results of Zhang *et al.* (2025 ▸), temperature-independent, constant material properties are used only in this section (Section 3.1), as well as the same average power of 262 W. The relation between the average power *P*_av_, pulse energy *Q*_p_, and repetition frequency *f*_rep_ is given by *P*_av_ = *Q*_p_*f*_rep_.

To quantify the thermal distortion of the crystal lattice plane, we use the thermal deformation of the crystal surface both in slope error and height error. The extinction length of the silicon and diamond crystal in our application (Bragg angle is about 45°) is around 10 µm (Shi *et al.*, 2023 ▸; Boesenberg *et al.*, 2019 ▸). The deformation of the crystal surface is directly related to the distortion of the top tenth micrometres crystal volume impacting the X-ray diffraction. Thermal deformation in slope error or height error is the relative surface deformation – differential surface shape with heat load and without heat load. This should be identical to the crystal plane thermal distortion within the extinction length.

**Figure 6:**
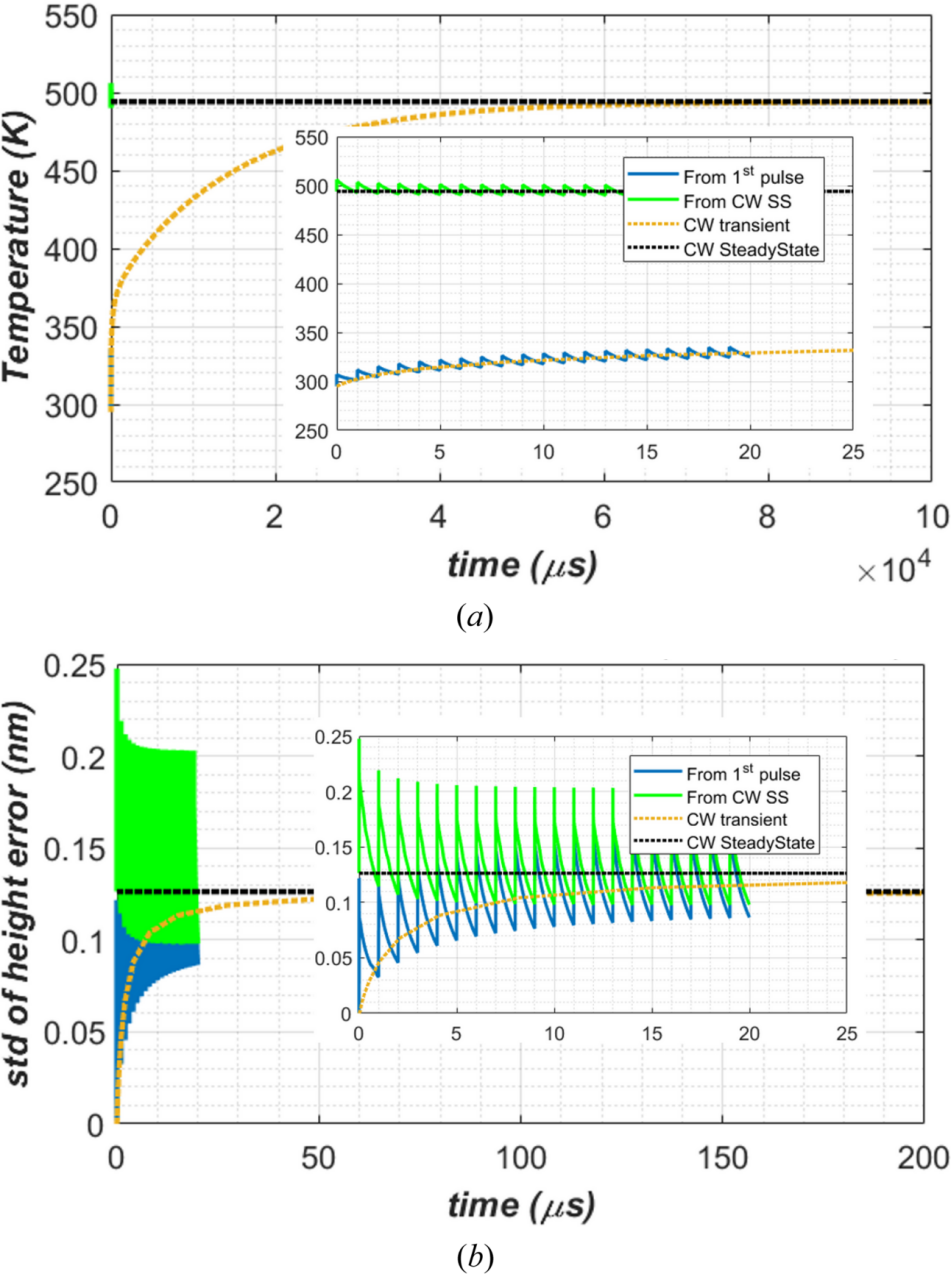
Pulse-by-pulse transient responses to the first XFEL pulses: (*a*) temperature at the centre of the footprint, (*b*) the standard deviation of the height error of the beam footprint on the diamond crystal over 2×FWHM length, 1 MHz repetition frequency, 0.262 mJ pulse energy. Two initial conditions were used in the simulation: (i) ‘From 1st pulse’, where the crystal was at room temperature, (ii) ‘From CW SS’, steady-state simulation results with average XFEL power. To guide the visualization, the results of steady-state and transient analyses with average power of 262 W (CW case) are also plotted in dashed lines.

For the CW case, the transient temperature and thermal deformation converge to the steady-state results in about 50 ms for temperature [Fig. 6 ▸(*a*)], and in about 50 µs for thermal deformation [Fig. 6(*b*) ▸]. Pulse-by-pulse full transient simulation results using uniform room temperature as the initial condition (‘From 1st pulse’) follow and oscillate around the results of the CW transient. This means that, to reach quasi steady-state, such pulse-by-pulse transient simulation should be conducted to about 50 ms of XFEL pulses, or 50000 pulses in 1 MHz repetition frequency. When using CW steady-state results as the initial condition (‘From CW SS’), pulse-by-pulse full transient simulation results converge quickly and vary around the results of the CW steady-state results. The convergence time is less than 10 µs. Therefore, using CW steady-state results as the initial condition, we can conduct the pulse-by-pulse full transient simulation for only a few XFEL pulses (*N*_p_) to reach quasi steady-state. The number of pulse *N*_p_ can be calculated by *N*_p_ = max(2, 10*f*_rep_) where repetition frequency *f*_rep_ is in MHz units.

## 4. Cryocooling and water cooling

In the following sections, we will exclusively use ‘From CW SS’ initial conditions, and temperature-dependent material properties both for LN_2_ and water cooling in all the pulse-by-pulse full transient simulations. The following beam parameters will be used: average XFEL beam power of 150 W, beam size of 100 µm FWHM, and X-ray photon energy of 9.831 keV which corresponds to diamond crystal (400) at a 45° Bragg angle. As mentioned by Tang *et al.* (2023 ▸) and Zhang *et al.* (2025 ▸), the majority of the FEL radiation spectrum is slightly switched out of the narrow reflection bandwidth of the diamond crystal for output; only a small portion of the spectrum within the crystal Bragg reflection width is recirculated to seed the next electron bunch. Therefore, the absorbed power by the 50 µm-thick diamond crystal at 45° is about 8.41 W.

We will use an effective convection cooling coefficient of *h*_cv-eff_ = 0.01 W mm^−2^ K^−1^ on the crystal cooled surfaces as in Zhang *et al.* (2025 ▸). Two cooling fluids will be considered: LN_2_ at 77 K and water at 295 K. We will compare the performance of both cooling cases.

**Figure 7:**
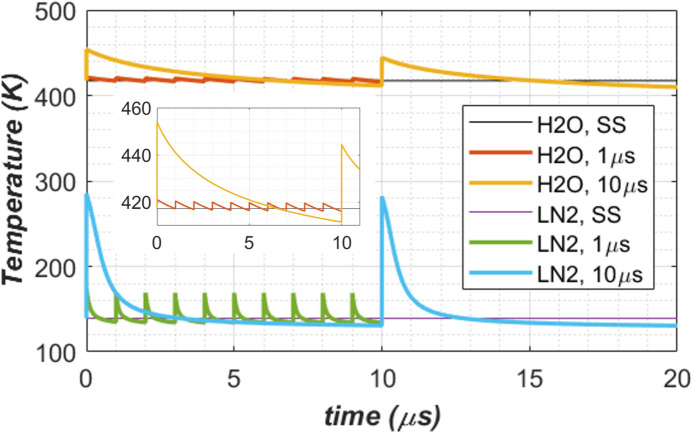
Temperature at the centre of the beam footprint versus time for repetition time *t*_per_ = 1, 10 µs, for both water (H_2_O) and liquid nitro­gen (LN_2_) cooling. Results from the CW steady-state (SS) are also plotted. A zoom plot of the water-cooling case is included.

Temperature at the centre of the beam footprint, or maximum temperature in the crystal, versus time is shown in Fig. 7 ▸ for both water (H_2_O) and LN_2_ cooling, and for CW steady state. Two repetition times *t*_per_ = 1, 10 µs were used for pulse-by-pulse transient analyses. The results from both water and LN_2_ cooling confirm that the steady-state (SS) case can be effectively used as the repetition time tends to zero, or repetition frequency tends to infinity, as explained by Zhang *et al.* (2025 ▸). With higher repetition frequency or smaller repetition time *t*_per_, the pulse energy is lower when keeping the same average power, the amplitude of the temperature oscillation around the results of the CW cases decreases. As expected, the temperature in the diamond crystal with LN_2_ cooling is lower than with water cooling. The sudden temperature increases with time before and after the XFEL pulse is higher with LN_2_ cooling than with water cooling, but the recovery time with LN_2_ cooling is shorter than with water cooling. All these behaviours are related to the material properties and are consistent with the discussion in Section 2.3. More quantitatively, we can calculate the temperature increase of the crystal related to the absorption of the XFEL pulse energy from the following equation,
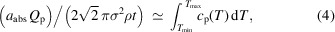
where *a*_abs_ is the absorption coefficient of the thin diamond crystal at 9.831 keV, which is equal to exp(−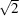 × 0.05/1.226) = 0.056; beam size in standard deviation σ = FWHM/2.35 = 0.04255 mm, crystal thickness *t* = 0.05 mm, density ρ = 2.53 × 10^−6^ kg mm^−3^; *T*_min_ and *T*_max_ are, respectively, the temperature at the centre of the footprint before and after the XFEL pulse. The 45° beam incidence was considered in equation (4) by the factor 1/sin(45) = 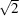. Table 2 ▸ compares the results of the sudden temperature increase determined by FEA and analytical calculations with equation (4). The estimates of the sudden temperature increase by equation (4) are in good agreement with the FEA results. The consistently lower temperatures calculated by equation (4) can be explained by the slightly variable in-depth power absorption in the diamond crystal which was considered in the FEA but averaged over the thickness of the crystal in equation (4).

At low temperature, a sudden heat input or XFEL pulse induces a much higher temperature increase of the crystals than at room temperature. This is, as indicated by equation (4), because the specific heat *c*_p_ (Fig. 4 ▸) at low temperatures is much lower than at room temperature. The temperature response just after the XFEL pulse might reach a higher peak with LN_2_ cooling than H_2_O cooling if the pulse energy is large enough. However, the thermal diffusion time at lower temperatures is much shorter than at room temperature due to much smaller thermal diffusion time (see Table 1 ▸). Therefore, thermal recovery is faster when the crystal is LN_2_ cooled than water cooled, despite higher instantaneous response just after the pulse.

**Figure 8:**
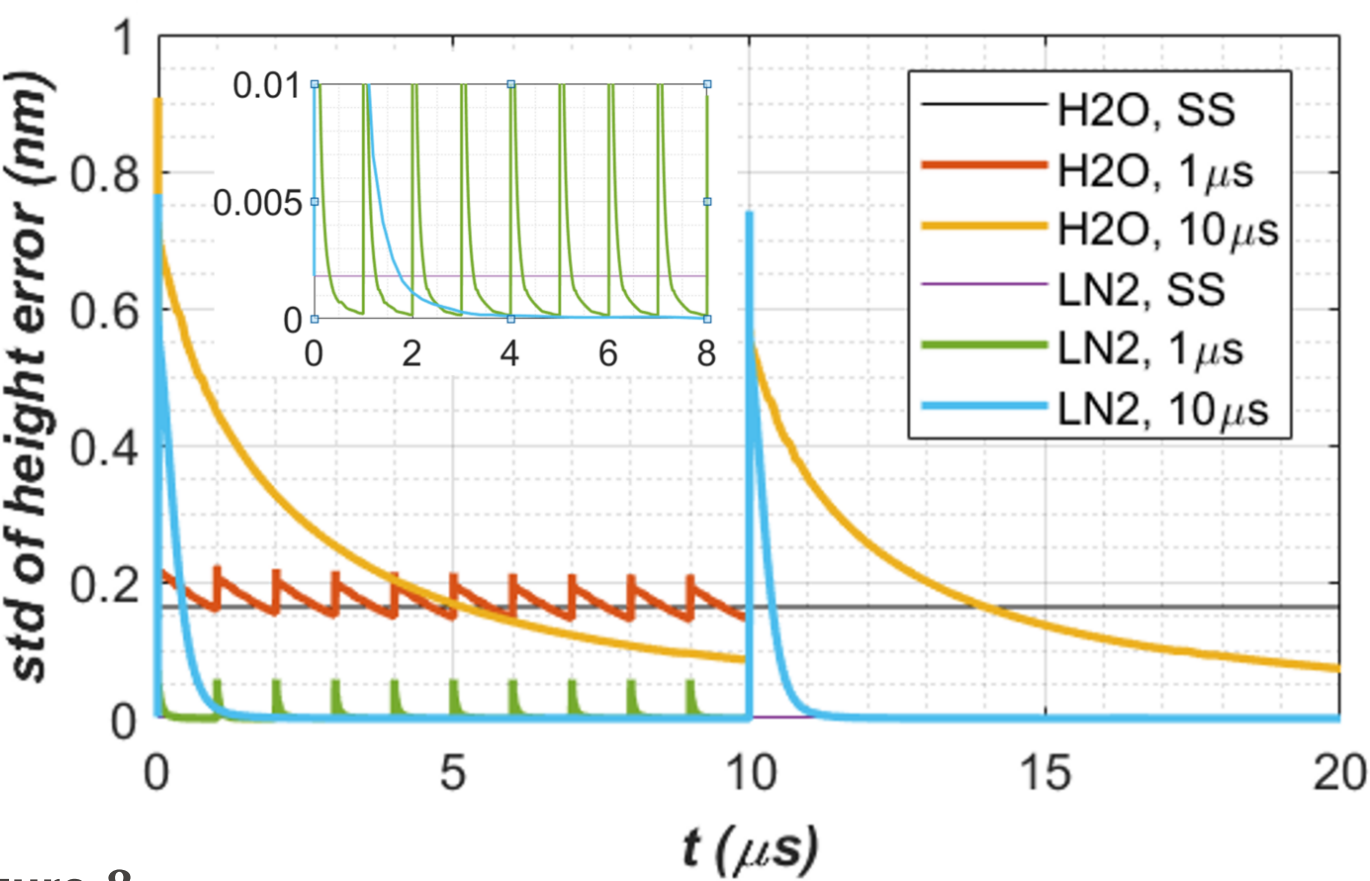
Standard deviation of height error along the beam footprint centre axis over 2×FWHM length versus time for repetition time *t*_per_ = 1, 10 µs, for both water (H_2_O) and liquid nitro­gen (LN_2_) cooling. Results from CW steady-state (SS) are also plotted. A zoom plot for the case of LN_2_ is included.

Thermal deformation in terms of the standard deviation of the height error along the beam footprint centre axis over 2×FWHM length is shown in Fig. 8 ▸. As thermal deformation is related to temperature gradient or temperature variation in the crystal, the thermal deformation generally exhibits similar behaviour as temperature (Fig. 7 ▸): (i) pulse-by-pulse transient results oscillate around the CW steady-state cases with smaller repetition time being closer to the CW case, for both water and LN_2_ cooling cases; (ii) thermal deformation is mostly lower with LN_2_ cooling than with water cooling; (iii) the sudden thermal deformation increases with time before and after the XFEL pulse is higher with LN_2_ cooling than with water cooling, but the recovery time of the LN_2_ cooling case is much shorter than the water cooling case. Point (iii) in thermal deformation is much more pronounced than in temperature. In fact, the peak thermal deformation in height error of the crystal cooled by LN_2_ can be higher than water cooling just after the XFEL pulse. This peak is due to the higher temperature raise related to the lower heat capacity of diamond crystal at LN_2_ temperature than at room temperature.

The short pulse FEL power absorption leads to a sudden spike in temperature and thermal deformation. To quantify in detail these observations, Table 3 ▸ compares results of the temperature at the centre of the beam footprint on the crystal surface *T*_c_ and the thermal deformation in terms of the standard deviation of height error along the beam footprint centre axis over 2×FWHM length std at three key moments: just after XFEL pulse power loading, at first-turn time 1 µs, and at period-end time *t*_per_. With repetition times *t*_per_ = 10, 50 µs, the thermal deformation with LN_2_ cooling just after the XFEL pulse can be higher than with water cooling. However, the recovery in thermal deformation with LN_2_ cooling is much quicker than with water cooling. For the case of *t*_per_ = 10 µs, with LN_2_ cooling the height error std from a peak value of 742 pm is reduced by a factor of 63 to 11.8 pm at the first-turn time of 1 µs, and by a factor of 1.5 × 10^5^ to 0.005 pm at the period-end time 10 µs. With water cooling the height error std from the peak value of 735 pm is reduced only by a factor of 2.1 to 356 pm at the first-turn time of 1 µs, and by a factor of 10 to 72.9 pm at the period-end time 10 µs. Compared with water cooling, thermal deformation with LN_2_ cooling is reduced by a factor of about 90 in the CW steady state case. Yet, in the pulse-by-pulse transient case, the LN_2_ cooling can lead to higher thermal deformation than water cooling just after the XFEL pulse. As the recovery time with LN_2_ cooling is much quicker than with water cooling, it is better to increase the first-turn time to maximize the benefits of LN_2_ cooling. That means a longer round-trip cavity. The higher the pulse energy, the longer the recovery time. Basically, when the temperature of the diamond crystal is below 200 K, the thermal properties become attractive for reducing thermal deformation.

For wavefront preservation requirements, the thermal deformation of the crystal in terms of standard deviation of height error should be smaller than 15 pm (Zhang *et al.*, 2023 ▸; Zhang *et al.*, 2025 ▸). In Table 3 ▸, the height errors smaller than these values are in bold. These results show that an LN_2_ cooled diamond crystal provides satisfactory performance in a 300 m round trip CBXFEL for repetition times of 10 µs or shorter and up to 150 W average XFEL power.

## 5. Second-order correction

**Figure 9:**
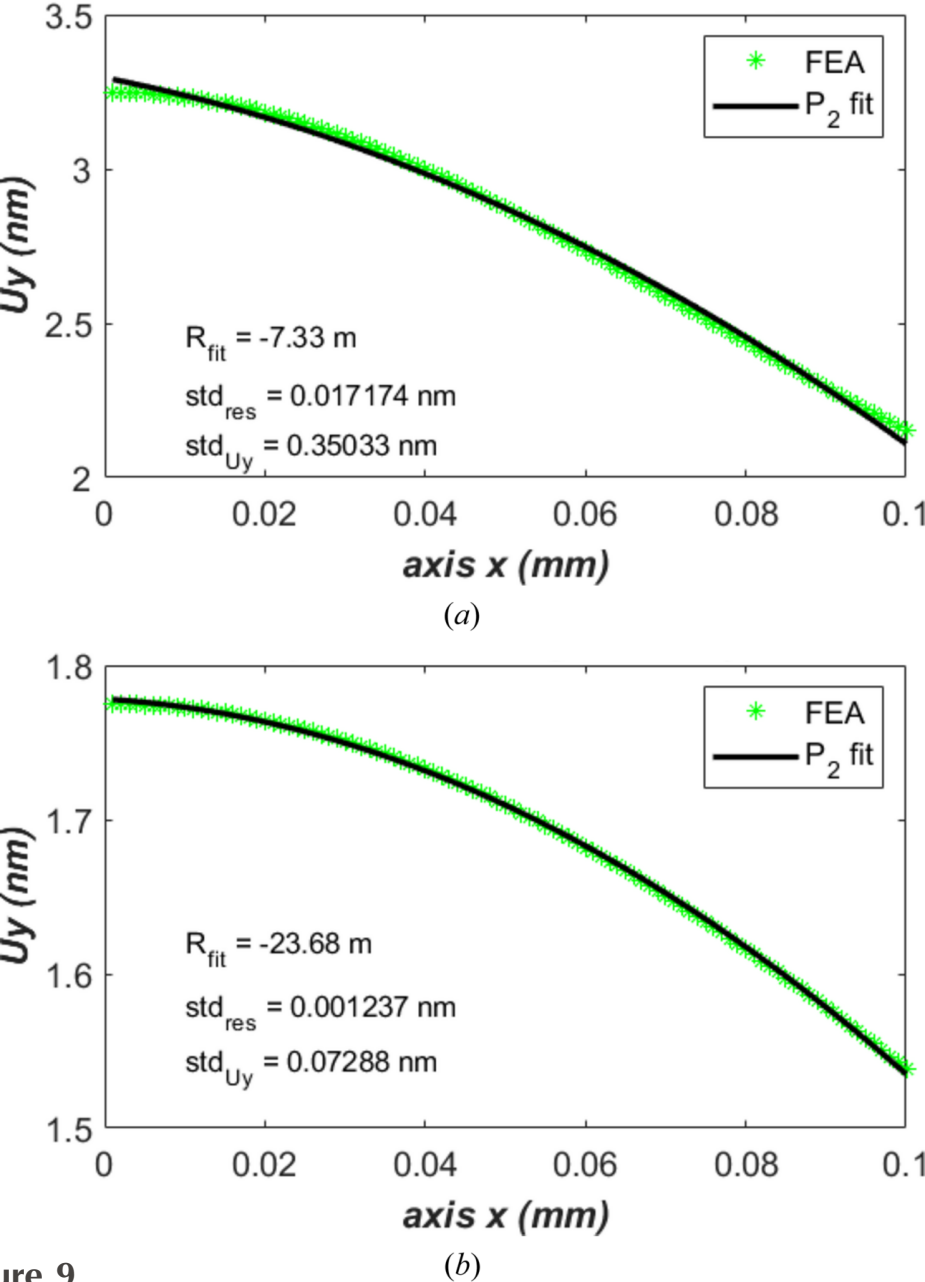
Vertical displacement *U*_*y*_ along the beam footprint and the best fit polynomial *P*_2_ for the case of water cooling, repetition time *t*_per_ = 10 µs: (*a*) the results at 1st turn time 1 µs after the XFEL pulse; (*b*) for the results at period end time 10 µs.

The cross-sectional profile of the power load on the crystal is a Gaussian distribution. This power profile leads to a thermal deformation shape that is nearly spherical. A focusing element within the cavity, for instance compound refractive lenses as shown by Fig. 1 of Zhang *et al.* (2025 ▸), can partially compensate for the effects of this spherically shaped thermal deformation. This spherical shaped compensation of the thermal deformed crystal is the so-called second-order correction. The vertical displacement *U*_*y*_ of the crystal along the beam footprint, the best fit polynomial *P*_2_, is shown in Fig. 9 ▸ for the case of water cooling, repetition time *t*_per_ = 10 µs: Fig. 9 ▸(*a*) for the results at first-turn time 1 µs after the XFEL pulse, and Fig. 9 ▸(*b*) for the results at period end time. The value of the residual, *i.e.* the difference between fit and FEA results, is indicated in Fig. 9 ▸, as well as the coefficient *a* = 1/2*R*_fit_ in front of THE *x*^2^ term in the *P*_2_ polynomial fit. These results show that the second order correction can reduce the thermal deformation by a factor greater than 20.

**Figure 10:**
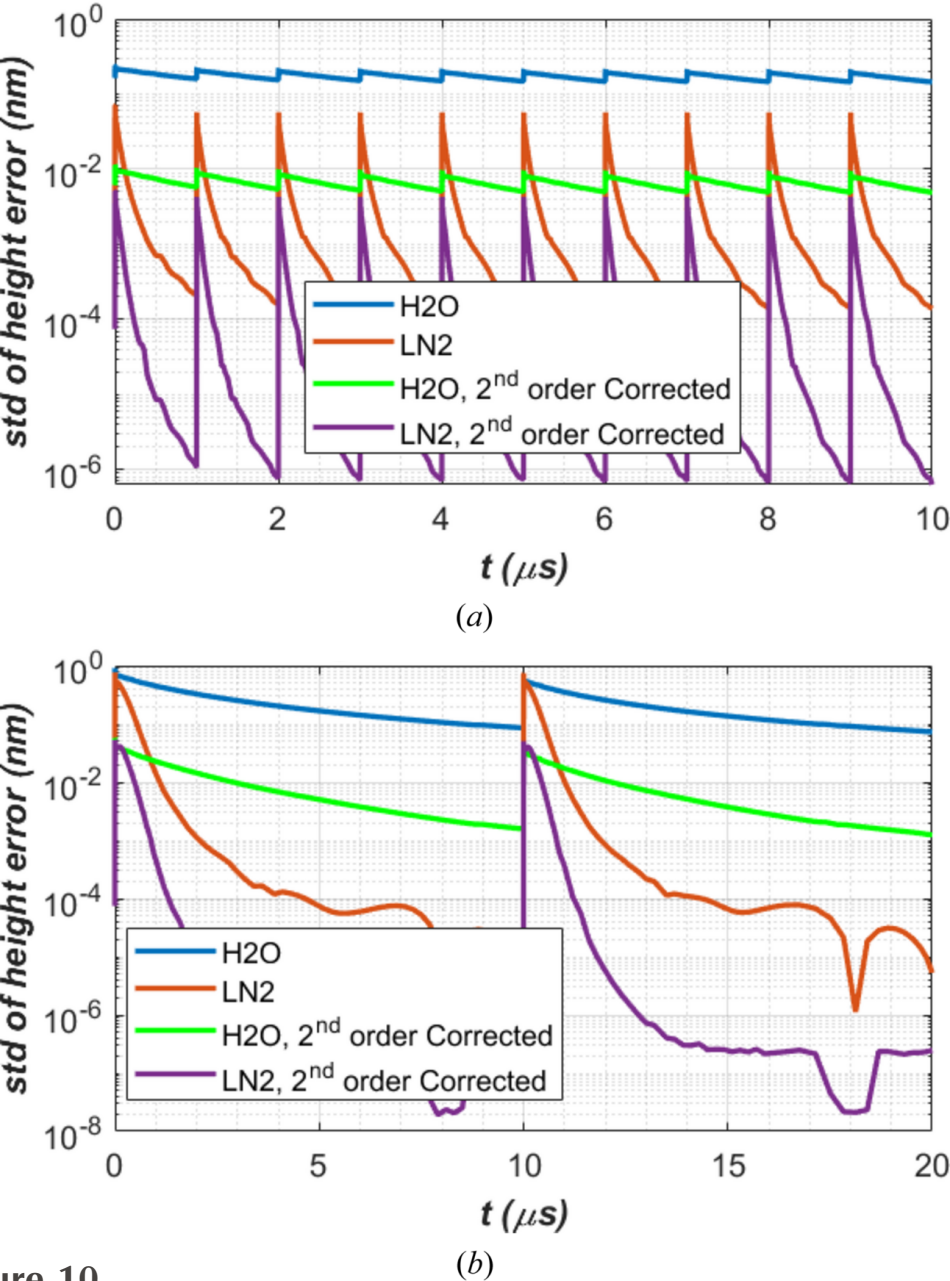
(*a*) Vertical displacement *U*_*y*_ along the beam footprint centre axis over FWHM length and second order polynomial fit at 1 µs after XFEL pulse; (*b*) the higher order deformation residual, *i.e.* the difference between fit and FEA results for the case of either water or LN_2_ and repetition time *t*_per_ = 10 µs.

For each time step, we can carry out this fitting operation and calculate the standard deviation of the residual. Results are plotted in Fig. 10 ▸ for both H_2_O and LN_2_ cooling, and for the cases of two repetition times *t*_per_ = 1, 10 µs. In general, LN_2_ cooling leads to a lower thermal deformation than H_2_O cooling 1 µs after the XFEL pulse, and second order correction reduces thermal deformation by a factor greater than 10. To quantify these effects, Table 4 ▸ compares results of the thermal deformation in terms of standard deviation of height error or residual along the beam footprint centre axis over 2×FWHM length at three key moments: just after XFEL pulse power loading, at first-turn time 1 µs, and at period-end time *t*_per_. The reduction factor *f*_reduction_ by second order correction is also given in Table 4 ▸.

For the diamond crystal in the 300 m round trip CBXFEL and 150 W average power, results in Fig. 10 ▸ and Table 4 ▸ all show that water cooling combined with second order correction can provide comparably acceptable performance results to LN_2_ cooling without second order correction: thermal deformation of the crystal is below the requirement (∼15 pm) for wavefront preservation for repetition time *t*_per_ ≤ 10 µs. When repetition time *t*_per_ ≥ 50 µs, the thermal deformation of the diamond crystal with a 150 W average power FEL is well above the requirement for wavefront preservation, including LN_2_ cooling with or without second order correction. In the repetition time and pulse energy, the turning point for acceptable performance is around *t*_per_ ≃ 20 µs and *Q*_p_ ≃ 3 mJ.

**Figure 11:**
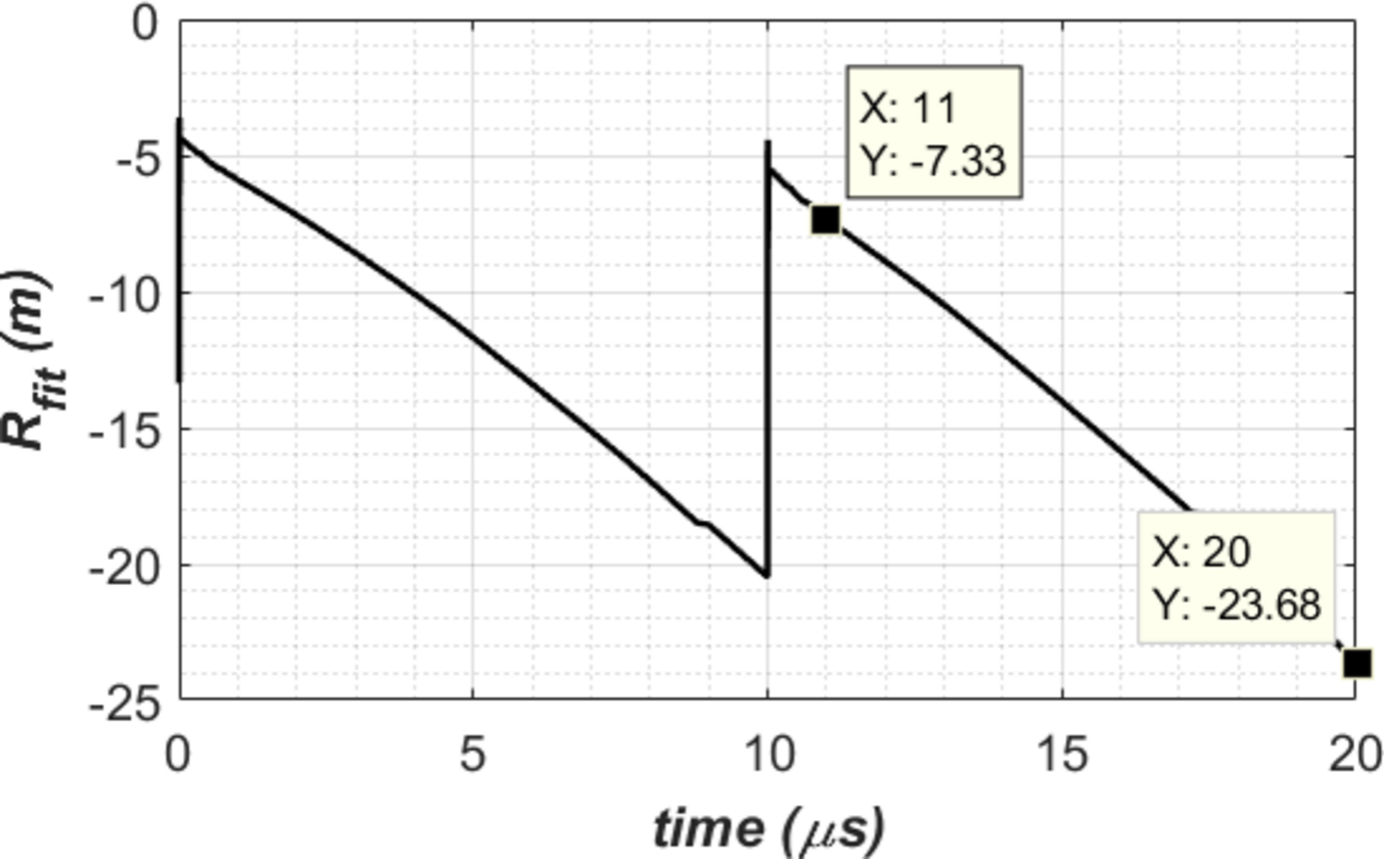
Second order correction coefficient in radius of curvature for the case of water cooling and repetition time *t*_per_ = 10 µs.

**Figure 12:**
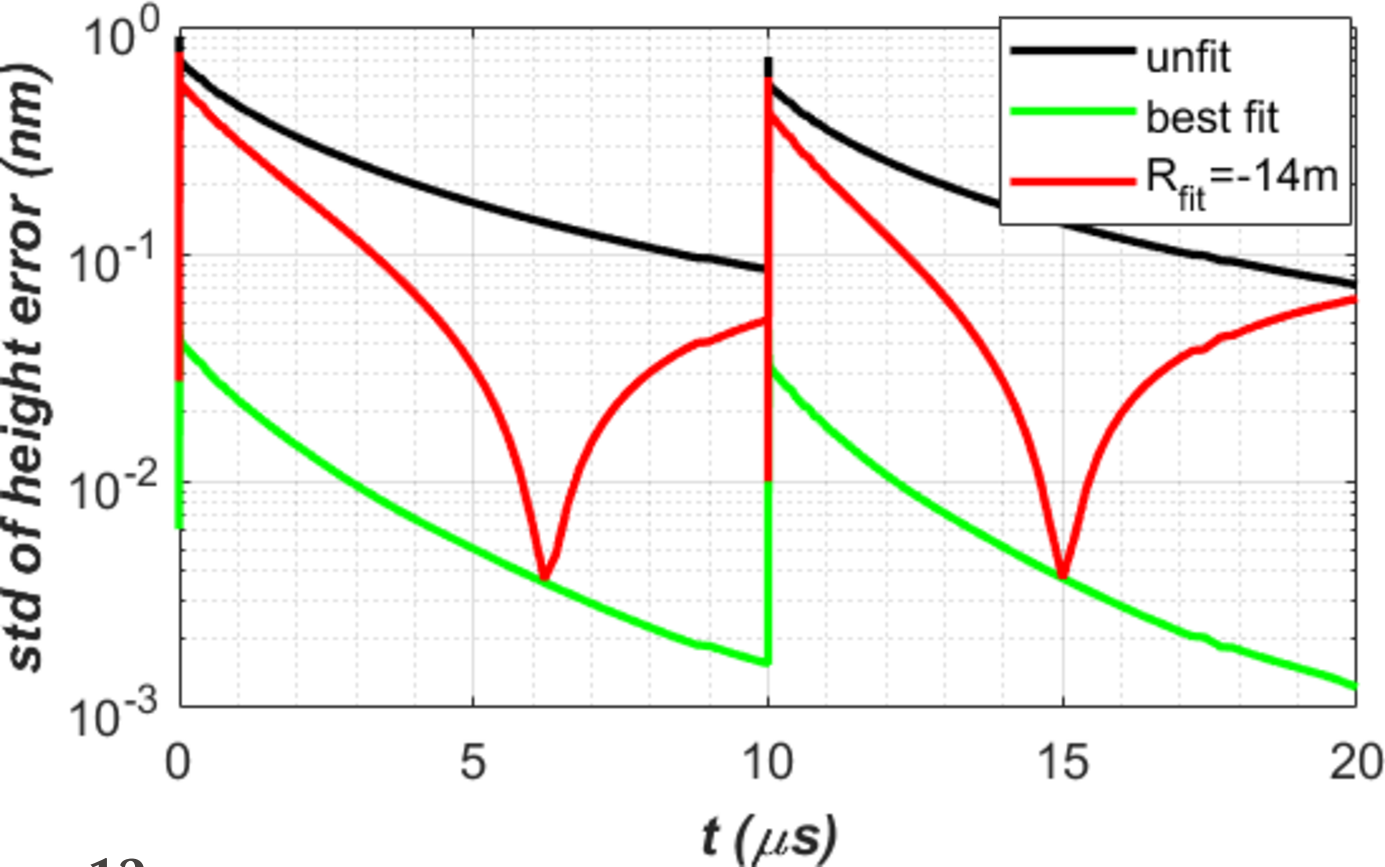
Thermal deformation in std height error for the case of water cooling and repetition time *t*_per_ = 10 µs.

The second order correction coefficient in terms of radius of curvature *R*_fit_ is plotted in Fig. 11 ▸ for the case of *t*_per_ = 10 µs and water cooling. The radius *R*_fit_ varies with time. It is necessary to make this shape correction when the recirculating FEL in the cavity hits the first diamond crystal C_1_ in the 300 m round trip CBXFEL. The corresponding time is at 1, 2,…, 10 µs after the arrival of the newly generated XFEL pulse. *R*_fit_ varies from −7.33 to −23.7 m. The negative sign means that the thermal deformation shape is convex or defocusing. To make this second order correction effective, the focusing element in the cavity should be able to provide a variable compensation focusing capability of 7.33 to 23.7 m in radius of curvature with a time resolution better than 1 µs to adjust the focusing range. Engineering challenges for such compensation using a dynamic focusing system are: (i) better than 1 µs time resolution for both shape detection and focusing optics actuation; (ii) accuracy of both detection and actuation should provide much better than 10 pm height error on the crystal. It is conceivable that a fixed focal length wavefront curvature compensation optics can be introduced within the cavity, *e.g.* to both partially compensate for an *R*_fit_ of −10 to −15 m, and to stabilize the beam orbit within the cavity at the same time (Margraf *et al.*, 2023 ▸). As an example, we use such optics for a fixed second order compensation *R*_fit_ = −14 m for the case of water cooling and repetition time *t*_per_ = 10 µs; results are compared with unfit and dynamically best fit as shown in Fig. 12 ▸. It is clear that a fixed focal length optics has partial wavefront curvature compensation effects.

## 6. Summary and conclusion

In this paper, we have revisited temperature dependent thermal mechanical properties of diamond crystal and presented the proposed polynomial form data fitting in the Appendices[App appa]. We proposed a method to accelerate convergence of pulse-by-pulse transient simulation using FEA results in the CW steady-state case as an initial condition. The convergence time is less than 10 µs, compared with 50 ms in temperature and 50 µs in thermal deformation starting from first pulse. Therefore, by using CW steady-state results as an initial condition, we can conduct the pulse-by-pulse full transient simulation for only a few XFEL pulses to reach quasi steady-state.

Concerning the temperature and thermal deformation of the crystal under CW average power, LN_2_ cooling reduces the temperature from about 410 K to roughly 130 K (see Fig. 7 ▸), and reduces the thermal deformation of the diamond crystal by a factor of about 80 (see Fig. 8 ▸), compared with water cooling. The difference between water and LN_2_ cooling in the case of pulse-by-pulse response is more complex than for the CW case. Immediately after a pulse, the amplitude of crystal temperature increases on the beam footprint with LN_2_ cooling can be higher than with water cooling, because of low specific heat of the diamond crystal at cryo-temperature than at room temperature. The thermal deformation of the crystal is proportional to the temperature gradient or temperature variation. The amplitude of the thermal deformation increases in the LN_2_ cooling case are also higher than in the water-cooling case. During FEL seeds recirculating in the cavity, the temperature of the crystal with LN_2_ cooling decreases much faster than with water cooling thanks to the much higher thermal diffusivity. Furthermore, the thermal expansion coefficient of the crystal tends towards zero at LN_2_ temperatures. Therefore, the thermal deformation of the crystal with LN_2_ cooling decreases much faster than with water cooling and reaches nearly zero; for instance, the standard deviation of the height error (std) reaches 0.1 pm at 1 µs repetition time and 0.01 pm at 10 µs repetition time, for 150 W average FEL power (see Fig. 10 ▸). Results show that LN_2_-cooled diamond crystals meet the stringent deformation requirement of less than 15 pm RMS for the pulse energy much larger than 0.15 mJ (probably 1 mJ) for 1 MHz repetition frequency, and up to 1.5 mJ for 100 kHz. The thermal deformation of an LN_2_ cooled diamond crystal is no longer a limiting factor for supporting high repetition FELs up to 300 kHz.

Second order correction by using focusing elements in the cavity can, indeed, reduce the thermal deformation for both LN_2_ and water cooling. However, dynamically adjustable focusing at better than 1 µs time resolution in the cavity is not trivial and challenging to engineer.

## Figures and Tables

**Table 1:** Thermal diffusivity, typical thermal diffusion time in a length of 1 mm

	Thermal diffusivity, *D* (m^2^ s^−1^)				Thermal diffusion time, τ_d_ (µs)		
*T* (K)	Si-nat	Si-28	Diamond		Si-nat	Si-28	Diamond
20	0.66	3.88	4.82		1.52	0.26	0.21
25	0.23	1.10	3.99		4.35	0.91	0.25
77	0.0035	0.0085	0.60		286	117	1.67
300	8.94 × 10^−5^	0.000147	0.00195		11186	6801	512

**Table 2:** Maximum and minimum value of temperature at centre of beam footprint on the crystal just before and after XFEL pulse power loading

		H_2_O cooling		LN_2_ cooling	
	*t*_per_ (µs)	1	10	1	10
	*Q*_p-abs_ (µJ)	8.42	84.2	8.42	84.2
FEA results	*T*_min_ (K)	416.1	411.5	134.0	130.8
	*T*_max_ (K)	419.5	444.5	168.9	282.0
	Δ*T*(max − min) (K)	3.4	33.0	34.9	151.1
Analytical results	*T*_max_ (K)	419.4	443.3	168.6	270.7
	Δ*T*_max_(FEA− Ana) (K)	0.1	1.2	0.3	11.2
	ɛ_(FEA−Ana)_ (%)	2.9	3.7	0.9	7.4

**Table 3:** Temperature at the centre of the beam footprint on the crystal *T*_c_, standard deviation of height error along the beam footprint centre axis over 2×FWHM length std at three key moments: just after the XFEL pulse power loading; at first-turn time 1 µs; at period-end time *t*_per_ Results are compared between water (H_2_O) and liquid nitro­gen (LN_2_) cooling, between three different repetition times *t*_per_ = 1, 10, 50 µs. Results for CW steady state analyses with either H_2_O or LN_2_ cooling are also included as reference. The reduction factor of the height error std by LN_2_ cooling related to H_2_O cooling *f*_reduction_ = std(LN_2_)/std(H_2_O). The same average XFEL power is assumed.

		H_2_O cooling		LN_2_ cooling		
Height error (pm)		*T*_c_ (K)	std (pm)	*T*_c_ (K)	std (pm)	*f*_reduction_ by LN_2_ cooling
*t*_per_ = 50 µs	Peak	543	3282	466	3778	0.87
	At 1 µs	512	1521	398	1826	0.83
	At *t*_per_	399	19.3	129	**0.02**	**1108**

*t*_per_ = 10 µs	Peak	444	735	282	742	0.99
	At 1 µs	434	356	164	**11.8**	**30**
	At *t*_per_	411	72.9	131	**0.005**	**13748**

*t*_per_ = 1 µs	Peak	419	210	169	56.8	3.7
	At 1 µs	416	147	134	**0.15**	**1008**
	At *t*_per_	416	147	134	**0.15**	**1008**
CW steady state		417	163	139	**1.82**	**89.5**

**Table 4:** Standard deviation of height error over the beam footprint 2×FWHM on the crystal surface at three times: just after XFEL pulse power loading (0.3–6 ns), first round trip time 1 µs, and period end time *t*_per_; for four different cases: water or LN_2_ cooling, and with or without second order correction. The second order correction factor is also shown here

		std height error (pm)					H_2_O	LN_2_
Height error (pm)		H_2_O	LN_2_	H_2_O, 2nd cor	LN_2_, 2nd cor		*f*_reduction_ by 2nd order correction	
*t*_per_ = 50 µs	Peak	3282	3778	220	258		15	15
	At 1 µs	1521	1826	92.6	188		16	10
	At *t*_per_	19.3	**0.02**	**0.1**	**0.0009**		195	21

*t*_per_ = 10 µs	Peak	735	742	45.5	50.8		16	15
	At 1 µs	356	**11.8**	17.6	**0.42**		20	28
	At *t*_per_	72.9	**0.005**	**1.24**	**0.0002**		59	21

*t*_per_ = 1 µs	Peak	210	56.8	**9.33**	**4.24**		22	13
	At 1 µs	147	**0.15**	**5.0**	**0.0008**		29	187
	At *t*_per_	147	**0.15**	**5.0**	**0.0008**		29	187

## Footnotes

1 A Windows workstation (Intel Xeon CPU E5-2687 W v4 @ 3.00 GHz, 12 cores, 160 GB RAM, 8 TB HD).

## Appendix A Data fit equations for diamond, natural and single isotope silicon

As explained in Section 2, thermal conductivity, thermal expansion coefficient and specific heat are temperature dependent. Data from references have been fitted by equations and fitted results have been plotted in Figs. 1 ▸, 2 ▸, and 4 ▸ for diamond crystal, natural silicon and single-isotope silicon-28. In our FEA simulations, we used these material properties calculated by fitted equations.

### A1. Thermal conductivity versus temperature

For natural silicon:

For single-isotope silicon-28:

For diamond IIa:

the thermal conductivity *k* in units of W mm^−1^ K^−1^, temperature *T* in K.

### A2. Thermal expansion coefficient

For silicon:

For diamond:

the thermal expansion coefficient α in units of K^−1^, temperature *T* in K.

### A3. Specific heat

For silicon:

For diamond:

the specific heat *C*_p_ in units of J kg^−1^ K^−1^, temperature *T* in K.
